# The interacting effects of irrigation, sowing date and nitrogen on water status, protein and yield in pea (*Pisum sativum* L.)

**DOI:** 10.1038/s41598-022-20216-5

**Published:** 2022-09-25

**Authors:** Abolfazl Ghodsi, Tooraj Honar, Bahram Heidari, Mahdiyeh Salarpour, Mohammad Etemadi

**Affiliations:** 1 Department of Water Engineering, School of Agriculture, Shiraz University, Shiraz, Iran; 2 Department of Plant Production and Genetics, School of Agriculture, Shiraz University, Shiraz, Iran; 3 Department of Horticultural Sciences, School of Agriculture, Shiraz University, Shiraz, Iran

**Keywords:** Plant ecology, Plant physiology, Plant stress responses

## Abstract

Management for agronomic practices might improves growth and grain yield in pea. The main objective of this experiment was to assess the interacting effects of different irrigation regimes, sowing date and nitrogen fertilizer treatments on pea traits. We evaluated three irrigation regimes (50, 75, and 100% of the plant irrigation requirement), two sowing dates (February and March), and nitrogen [application of nitroregn (N1) and without nitrogen as control (N0)] in 2019 and 2020 under field conditions. Chlorphyll content, leaf area index, leaf water potential, grain yield and water productivity were higher in the late sowing (March) than in early sowing (February) treatment. Percentage of vegetation cover in late sowing (60%) was significantly higher than in early sowing (52.7%) treatment. Grain yield in 75% water requirement treatment was not significantly different from yield in full irrigation treatment. Application of nitrogen fertilizer significantly reduced grain yield, grain protein and seeds per pod whilst increased chlorophyll content only. The 100% irrigation requirement treatment showed higher evaporation form the soil in N0 than in 50% and 75% irrigation treatments in late sown pea. Leaf evapotranspiration (ET) was lower in 50% water requirement irrigation regime than in the other irrigation treatments. Water use efficiency (WUE) which was higher in the late than early sowing treatment did not differ between 50% and full irrigation treatments in N0. In conclusion, the results of the current study suggested that application of nitrogen fertilizer did not benefit pea growth and that management of irrigation regime in late sowing might improve grain yield in pea and save irrigation water in regions with limited water availability.

## Introduction

The pea (*Pisum sativum* L.) is an important legume ranked fourth in the total legume production worldwide^1^. This edible product is a part of humans diet in the middle and southeast Asia^2,3^. The increasing concern about personal health, quality, and security of the food that is related to the tendency to vegetarianism affects pea consumption worldwide. The pea seed richs in protein (39.75%) and has mixed carbohydrates, vitamins, minerals, and anti-oxidant ingredients^4,5^. Regular consumption of pea in human diet helps health and reduces the risk of cancer^6^.

The growth cycle of the pea is relatively short which requires lower water supply compared with other semi and broad-leafed crops^7,8^. Development of various agronomic managements to cope with the adverse effects of climate changes on crop production is one of priorities in sustainable agriculture. Water scarcity is one of the most important challenges for pdocution in agriculture. Irregular irrigation results in varation in size, shape, color, and maturity time in pea^9^. However, one or more supplemental irrigations at the beginning of the flowering and filling pods stages increase grain yield. The results of Rasaei et al.^10^ showed that the effects of irrigation on the green pod and the biological performance were significant in pea. Although nitrogen is one of the most important pollutants of water and air, management for nitrogen use migh improve plant growth and yield^11^. The effects of nitrogen fertilizer on grain yield and yield components of legume crops have been studied in crop plants^11–13^, but less has be devoted to analyze the influence of nitrogen in pea under field conditions. The rate of biological nitrogen fixation in pea is around 150 kg ha^−1^ that shows pea is adapted to cultivation under variable soil nitrogen contents^14,15^. Hu et al.^16^ have shown that low nitrogen application increases nitrogen concentration in pea. In a study, nitrogen improved the development of the plant leaf area by affecting the size and longevity of the leaf. A higher leaf area index that can help higher nitrogen storage improves vegetative growth status of the plant and consequently a higher grain yield^17^. In low nitrogen soils, pea helps to stabilize nitrogen, release a large amount of free fertilizer to the soil and consequently increase in grain protein^18^. In a study in pea, analysis of the effects of methyl nitrogen on biomass dispersion between stem and roots indicated that 400 kg ha^−1^ nitrogen reduced the number of pods, number of leaves and stem length but increased root growth and root density^8^.

Decision for a proper sowing time might prevent the adverse effects of early season freezing and helps to avoid end season high temperatures in crop cycle^19^. Shifiting sowing date is an option for better agronomic management, crop stablishment and better growth in pea. In a study, grain yield in an autumn cultivation was around 1830 kg ha^−1^ higher than yield in pea cultivated in spring^20^. Resuls of Ahmed et al.^21^ study have shown that seed yield and yield contributing traits were higher in early sowing (13 November) than late sowing (28 November) of pea in Bangladesh. The results of Istiaq et al.^22^ study showed that sowing date had a significant effect on pea growth. Decision for sowing time in pea requires a compromise between being early enough to avoid end season drought and late enough to avoid other environmental stresses. Fuerthremore, crop-specific management for irrigation strategies ensure available water is used efficiently to meet a specific crop’s water requirements for maximum water productivity. Considering the importance of agronomic managements, the objective of the present study was to assess the interacting effects of moisture regimes, sowing date and application of nitrogen on growth parameters, water use efficiency (WUE) and yield related traits in pea.

## Materials and methods

### Field experiment and treatments

The plant sample that was not considered as threatened species and not listed as species with small or very small populations in Iran, was identified in the Laboratory of Department of Horticultural Scinces following the NCBI Taxonomy description (https://www.ncbi.nlm.nih.gov/Taxonomy/Browser/wwwtax.cgi?lvl=0&id=4545). The source of plant materials with the voucher ID of P-WLF-1401 was deposited in the Seed Bank of Department of Plant Production and Genetics, School of Agriculture, Shiraz University, Iran and are available for research purposes. All experiments including the collection and use of plant samples were conducted according to the relevant institutional, national, and international guidelines and legislation.

The experiment was performed in the 2018–2019 and 2019–2020 seasons in a silty- clay- loam soil at the School of Agriculture, Shiraz University, Shiraz, Iran. Soil characteristics are shown in Table 1. Prior to sowing, 100 kg ha^−1^ P_2_O_5_ was added to the soil. The layout of the experimental design presented in Suppl. Fig. 1 shows irrigation (I), sowing date (D) and nitrogen (N) treatments applied in the field. The experimental design was split split plot with three replicates. The main factor was irrigation with 50%, 75% and 100%, field capacity (FC). The seeds of the “Wolf” variety that was provided by the seed compay Emanuel Larosa, Italy, were sown on two sowing dates including February 20 and March 11 as sub-plots. The sub sub-plot was nitrogen treatments (with and without nitrogen). Each experimental plot included four 2.5 m long rows spaced 50 cm apart. The plant density was 160,000 seeds ha^−1^. Weeds were controlled manually.

**Table 1:** Characteristics of soil in different depths (cm) in the field in pea experiment.

Soil characteristics	0–10 (cm)	10–30 (cm)	30–60 (cm)	60–90 (cm)	90–120 (cm)
${\theta }_{FC }$ (cm^3^/cm^3^)	0.30	0.32	0.33	0.33	0.33
${\theta }_{pwp}$ (cm^3^/cm^3^)	0.16	0.16	0.19	0.19	0.19
*ρ*b (g/cm^3^)	1.30	1.43	1.43	1.43	1.43
Clay (%)	35.00	31.00	39.00	34.00	29.00
Silt (%)	55.00	57.00	51.00	50.00	53.00
Sand (%)	10.00	12.00	10.00	16.00	18.00
pH			7.8		
Tortal nitrogen			0.012%		
Available potassium			25 mg/kg		
Available phosphorous			12 mg/kg		

ρb, bulk density.

The amount of irrigation water for different irrigation treatments was determined using an auger device based on soil moisture measured at three soil depths of 0–20, 20–40, and 40–60 cm (Table 1). Evaporation from the soil surface was quantified using microlysimeters with PVC pipes of 10 cm diameter and 30 cm height. A metal mesh was placed in the bottom of the pipes that was applied in the stream before applying the treatments. Microlysimeters weighed for at least 24 h prior and after each rainfall and irrigation practice. Evaporation from 30 cm soil depth was calculated as follow,

$$\frac{{{\text{w}}_{2} - {\text{w}}_{1} }}{{{\uppi }{\text{r}}^{2} }}$$

where w1 and w2 were microlysimeters weights prior to and after irrigation, respectively and πr^2^ is cross section of evarpration (π is 3.14 and r is 5 cm).

### Phenotyping

Number of pods per plot, maximum grain per pod (MGP) and grain yield per plant (g) were measured at the physiological maturity in ten plant samples. The Kjeldahl method was used to measure total nitrogen and grain protein content (GPC)^23^. In this method, nitrogen was oxidized to concentrated ammonium sulfate with the help of concentrated sulfuric acid. Then, nitrogen was collected as ammonia gas by distillation. First, 1 g dried sample was wrapped in filter paper and 20 ml sulfuric acid was added. Then, 2 g catalyst added to the samples and the tubes were placed in a digester at 50 °C. The temperature gradually reached 250–300 °C. After 24 h, nitrogen (%) of the samples was measured using a Kjeldahl device. The GPC calculated as follows,

$$GPC=Kp\times GNC$$

where GNC is the nitrogen concentration of grain, *Kp* is the conversion coefficient of nitrogen to protein which is approximately 5.7 for legumes^24^.The plant evapotranspiration (ET, mm) was calculated by the water balance method as follows,

$$ET=I+P- D \pm \left(\sum_{i=1}^{n}( \theta 1i- \theta 2i)\Delta Zi\right)$$

where *I* is the irrigation depth (mm), *P* is the precipitation (mm), *D* is the deep percolation (mm) from the bottom of the root zone, *n* is the number of soil layers, *zi* is the thickness of each soil layer(mm) and $\theta$ 1i and $\theta$ 2i are the volumetric soil water contents before the consecutive wetting event in the same soil layers (cm^3^ cm^−3^).

Water use efficiency (WUE, kg m^−3^) was measured as grain yield per unit of water use as follows,

$$WUE = {Y \mathord{\left/ {\vphantom {Y {ET}}} \right. \kern-\nulldelimiterspace} {ET}}$$

where Y is grain yield (kg ha^−1^).

Water productivity (WP, kg m^−3^) was obtained by dividing grain yield by the amount of applied irrigation water (*Ig*, mm) as follows,

$$WP = {Y \mathord{\left/ {\vphantom {Y {Ig}}} \right. \kern-\nulldelimiterspace} {Ig}}$$

Leaf water potential and chlorophyll content were measured using pressure chamber (PMS Instruments Albany, OR, USA) and SPAD device (Minolta SPAD-502), respectively.

The leaf area was measured using a leaf area meter (SYSTRONICS, Leaf Area Meter-211) and leaf area index (LAI) was calculated as follow,

$$LAI=C{\sum }_{k=1}^{n}LiWi/AB$$

where, *C* is the crop coefficient, *Li* and *Wi* are leaf length and width (cm), *A* and *B* are the distance between two neighboring plants and rows distance (cm) in the field.

### Statistical analysis

The analysis of variance (ANOVA) of traits was performed in SAS software^25^. In the generalized lenear model (GML) model, treatment and year effects defined as fix and random, respectively. The means of the tested traits were statisticaaly compared based on the least significant differences (LSD) test. Main effects of treatments were not compared for traits with significant interaction between treatments. Slicing interaction method was used for mean comparison of traits with significant interaction between treatments. In slicing interaction analysis, levels of each treatment were statistically compared per each level of the others. For traits with non-significant irrigation × year and planting date × year interactions, the data averaged over years for mean comparisons.

## Result

### Yield related traits

**Figure 1:**
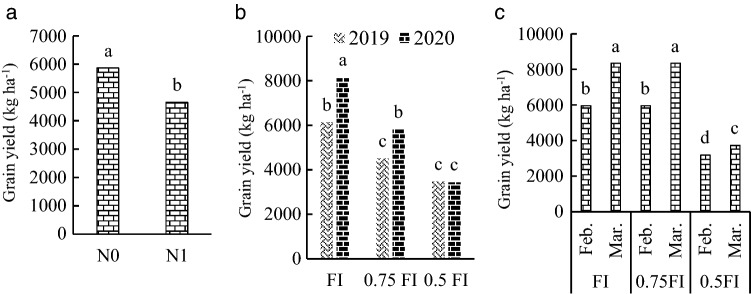
The effect of nitrogen fertilizer averaged over irrigation and sowing date (**a**), irrigation averaged over nitrogen and sowing date (**b**), and irrigation × sowing date interaction (**c**) on grain yield (kg ha^−1^) in pea. FI: full irrigation, N: nitrogen, Feb: February, Mar: March. Means with different letters did differ significantly.

The results of ANOVA showed that the quadruple interaction of irrigation × planting date × nitrogen × year were not-significant for grain yield, grain yield per plant, 100-grain weight, number of pods per plot and seeds per pods (Table 2). However, mean comparison was performed for significant effects comprising of nitrogen, irrigation × year and irrigation × sowing date. In the full irrigation (FI) treatment, grain yield increased by 36.14% and 40.5% compared to 0.75FI in 2019 and 2020, respectively. Grain yield in without nitrogen (N0) treatment was significantly higher than N1 treatment (Fig. 1a). Grain yield in FI and 0.75FI did differ signicicantly between 2019 and 2020 seaons (Fig. 1b). Highest grain yield that achieved in FI in the late sowing treatment did not differ significantly with grain yield in 0.75FI treatment (Fig. 1c).

**Table 2:** Combined analysis of variance of irrigation regimes, sowing date, nitrogen fertilizer and year effects for pea traits.

Source	DF	Chlorophyll content	Vegetation cover (%)	Leaf water potential	Plant evapotranspiration	Leaf area index	Soil evaporation	Grain protein	Grain yield per plant	100-grain weight	Grain yield	Water use efficiency	Water productivity	Number of pods per plot	Maximum grain per pods
Year (Y)	1	0.0001	1283.56**	0.13	32,669.94**	2.70**	53.07**	13.92**	33,555**	302.02**	21,428,759**	0.12	1.29**	4161.27**	5.63**
Error Y	4	39.10	70.10	0.02	170.32	0.08	0.07	0.80	23.70	10.64	75,192.00	0.02	0.01	46.65	0.05
Irrigation (I)	2	16.72	1095.01**	3.22**	303,875.11**	13.04**	0.58**	33.21*	6490.53**	2099.66**	82,100,848**	0.20	0.67**	2059.54**	21.04**
Y*I	2	0.00	0.93	0.2	1283.90**	0.52**	0.14	0.79	42.02	500.49**	6,488,499**	0.38	0.37**	20.92	1.23**
Error Y*I	8	21.85	161.18	0.04	134.826	0.05	0.10	0.59	131.46	7.95	355,936.00	0.04	0.03	241.61	0.07
Planting date (D)	1	188.18**	938.89**	0.03	8473.85**	0.76**	0.37	0.004	4747.59**	1674.41**	36,409,338**	1.55**	7.50**	889.81*	3.946**
I*D	2	20.18	15.26	0.28**	326.01**	0.04	2.04**	0.16	16.88	244.39**	5,138,101**	0.13*	0.18**	10.18	0.33
Y*D	1	0.00	14.22	0.00	743.05**	0.00	0.98**	0.73	117.23	81.28*	67,249.00	0.00	0.00	1.97	0.13
Y*I*D	2	0.00	8.85	0.02	41.11**	0.13*	1.25**	0.19	454.58**	68.09*	379,251.00	0.03	0.02	264.61	0.381*
Error Y*I*D	12	47.18	85.35	0.03	257.53	0.05	0.20	0.51	42.93	7.99	195,625.00	0.03	0.02	108.95	0.23
Nitrogen (N)	1	343.22**	133.39	0.23*	0.10	0.84**	0.06	12.69**	232.03*	2407.48**	26,517,001**	2.85**	2.45**	8.76	5.85**
I*N	2	95.71	1.93	0.09	1.75	0.06	0.07	1.36*	51.34	57.34*	139,549.00	0.157*	0.10*	143.40	0.20
D*N	1	26.89	2.72	0.02	0.62	0.00	0.42**	0.26	3.60	8.45	299,801.00	0.03	0.12*	101.99	0.02
I*D*N	2	0.00	1.35	0.01	2.19	0.03	0.14	0.12	71.94	78.42*	588,118.00	0.06	0.09	52.85	0.11
Y*N	1	0.00	6.72	0.02	3.25	0.17*	0.00	1.0	9.91	14.91	101,377.00	0.01	0.00	111.64	0.16
Y*I*N	2	25.45	6.01	0.04	1.01	0.00	1.42**	0.64	342.475*	117.81**	963,672.00	0.07	0.06	78.05	0.04
Y*D*N	1	0.00	0.06	0.12	2.61	0.03	0.01	0.47	6.46	1.81	25,119.00	0.00	0.00	34.49	0.05
Y*I*D*N	2	0.00	0.10	0.00	0.80	0.02	3.71**	0.23	125.18	2.61	6091.47	0.00	0.00	32.51	0.09
Error	24	29.97	50.56	0.05	5.50	0.03	0.09	8.17	45.96	14.34	325,326.00	0.04	0.03	168.44	0.10
Coefficient of variation (%)		10.96	12.60	10.87	0.69	5.73	0.19	11.41	10.43	5.21	10.84	12.62	11.19	12.97	6.03

Grain yield per plant was significantly affected by triple Y × I × D and Y × I × N interactions (Tables 3, 4). Mean comparison for Y × I × D showed that the highest grain yield per plant was achieved in 0.5FI in late sowing (108.93 g) of 2020 which was not significantly different with grain yield in the same treatment (100.67 g) in 2019 (Table 3). Results of mean comparison for Y × I × N interactions indicated that grain yield per plant in N1 -0.75FI (85.79 g) combined treatment was not significantly different from grain yield in N1-FI (106.25).

**Table 3:** The effects of irrigation regime and sowing date on pea traits tested in 2019 and 2020.

Season	Irrigation treatment	Sowing date	Grain yield per plant (g)	Maximum grain per pods	100-grain weight (g)
2019	FI	February	27.43 a	4.18 a	62.77 a
		March	56.97 abcde	4.3 ab	84.65 de
	0.75FI	February	32.61 ab	4.55 abcd	68.06 abc
		March	73.82 bcdef	5.44 abcde	79.03 bcde
	0.5FI	February	49.55 abcd	5.68 bcde	63.47 a
		March	100.67 f	6.09 ef	65.93 ab
2020	FI	February	32.47 ab	4.54 abcd	81.95 cde
		March	83.21 cdef	4.49 abc	92.37 e
	0.75FI	February	47 abc	4.99 abcde	73.27 abcd
		March	95.69 ef	5.82 cdef	81.09 cde
	0.5FI	February	71.18 bcdef	6.03 ef	57.74 a
		March	108.93 f	7.19 f	62.07 a

FI, full irrigation. The data averaged over nitrogen treatments, Means with different letters did differ significantly.

**Table 4:** The effects of irrigation regime and nitrogen fertilizer on grain yield per plant, water use efficiency and water productivity in pea.

	2019						2020					
	FI		0.75FI		0.5FI		FI		0.75FI		0.5FI	
	N0	N1	N0	N1	N0	N1	N0	N1	N0	N1	N0	N1
Grain yield per plant (g)	60.86 c	59.86 c	34.12 ab	45.49 b	28.64 a	31.27 a	103.36 d	106.25 d	83.72 bc	85.79 cd	68.3 a	71.88 ab
100-grain weight (g)	77.78 d	69.64 c	83.49 e	63.59 b	69.4 a	60.01 a	91.24 e	83.08 d	81.4 d	72.96 c	67.59 b	52.22 a

FI, full irrigation; N, nitrogen. Means with different letters did differ significantly.

**Figure 2:**
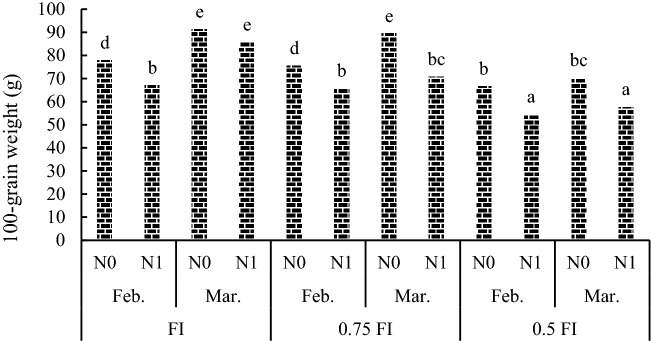
The effect of irrigation × sowing date × nitrogen fertilizer interaction on 100-grain weight (g) averaged over years in pea. FI: full irrigation, N: nitrogen, Feb: February, Mar: March. Means with different letters did differ significantly.

Results of ANOVA revealed that 100-grain weight was significantly affected by Y × I × D, I × D × N and Y × I × N interactions (Table 2). Mean comparison for I × D × N effects showed that the highest 100-grain weight achieved in the FI -Mar-N0 treatment combination did not differ significantly with 100-grain weight in FI-Mar-N1 and 0.75FI-Mar-N0 treatments (Fig. 2). The 100-grain weight trait in 0.5FI-Feb-N1 was not significantly different with 0.5FI-Mar-N1 treatment. Results of Y × I × N analysis showd that 100-grain weight (52.22 g) in 0.5FI × N1 in 2020 did not significantly differ with 0.5FI-N0 treatment (Table 4). In both years, FI-N0 and 0.75FI-N0 treatment combinations presented higher 100-grain weight compared with other treatments (Table 4).

The nitrogen main effect and triple interaction of I × D × Y were significant for maximum seeds per pod (MSP). The MGP trait in N0 treatment (5.55) did differ with MGP in N1 (4.99) (data not shown). The highset MGP achieved in 0.5FI-Mar treatmen combination was not significantly different from MGP in 0.75FI-March in both years (Table 3). For MGP, both 0.5FI and 0.75FI treatments in late sowing showed no significant differences with 0.5FI treatment in early sowing in 2020.

Interactions of treatments were not significant for the number of pods per plot (Table 2). Mean comparison for irrigation regime and sowing date main effects showed that pea in the early date and FI had higher pods per plot than in late sowing and other irrigation treatments, respectively. The maximum number of pods per plot achieved in FI (341.47) was significantly different from 0.75FI and 0.5FI treatments.

### Leaf and soil related traits

**Figure 3:**
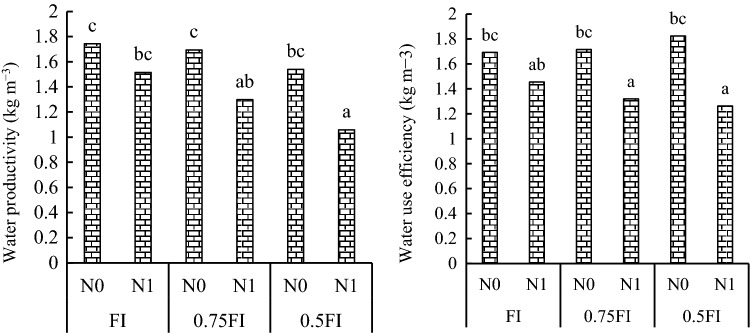
The effect of irrigation × nitrogen fertilizer interaction on water productivity (**A**) and water use efficiency (**B**) in pea. FI: full irrigation, N: nitrogen. The data averaged over sowing date and two years. Means with different letters did differ significantly

Water use efficiency (WUE) was affected by I × D, I × N and Y × I interactions (Table 2). The results showed that N0 treatment presented higher WUE than N1 in the three irrigation regimes. The highest WUE was obtained in the 0.5 FI- N0 treatment (1.82 kg m^−3^) which was not significantly different from WUE in 0.75FI moisture regeimes in N0 treatment (Fig. 3). Mean comparison for I × D interactions showed that WUE in the FI-Mar treatment combination (1.79 kg m^−3^) averaged over nitrogen treatments and years was not significantly different from 0.75FI-Mar (Table 5).

**Table 5:** The effects of irrigation regime and sowing date on water related traits in pea.

Traits	Sowing date	Irrigation regime		
		FI	0.75FI	0.5FI
Leaf water potential (Mpa)	February	1.73 a	2.07 b	2.24 bc
	March	1.55 a	2.13 bc	2.49 d
Water use efficiency (kg m^−3^)	February	1.36 a	1.36 a	1.47 ab
	March	1.79 c	1.67 bc	1.61 bc
Water productivity (kg m^−3^)	February	1.24 a	1.14 a	1.08 a
	March	2.02 d	1.85 c	1.52 b

FI, full irrigation. The data averaged on nitrogen and years. Means with different letters did differ significantly.

Irrigation and sowing date effects were significant for the percentage of vegetation cover (Table 2). Full irrigation treatment resulted in a 6% and 27% increase in vegetation cover compared with 0.75FI and 0.5FI, respectively. Percentage of vegetation cover in March sowing treatment (60%) was significantly higher than sowing in Feb (52.7%). Maximum vegetation cover (62.59%) observed for FI was significantly different from the coverage in 0.75FI and 0.5FI treatments.

**Figure 4:**
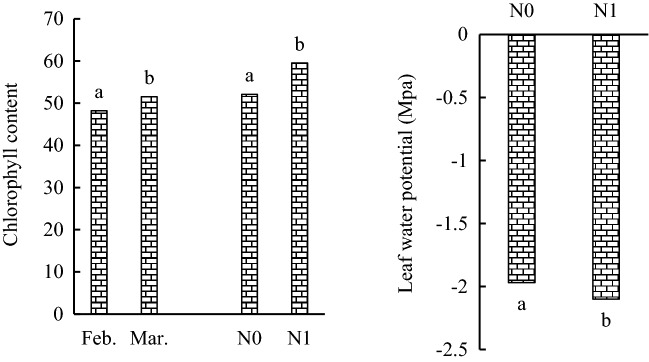
The effect of sowing date and nitrogen on the chlorophyll content (**A**), the effect of nitrogen of leaf water potential (**B**) in pea. N: nitrogen, Feb: February, Mar: March. Means with different letters did differ significantly.

The results of the analysis of variance showed that interaction of fertilizer and sowing date was significant for leaf chlorophyll content (Table 2). Chlorophyll content varied between 52.1 to 59.7 and 48.2 to 51.52 in nitrogen and sowing date treatments, respectively. The highest chlorophyll content was obtained in N1 with an average of 59.53, which was 14% higher than chlorophyll in N0 treatment (Fig. 4). Chlorophyll content in the late sowing treatment was higher than in early sowing. Leaf water potential (LWP) was affected by nitrogen main effect and I × D interaction (Table 2). Leaf water potential in the 0.5FI of late sowing treatment was significantly higher than LWP in other treatments (Table 5). Full irrigation treatment had lowest LWP in both sowing dates. In fertilizer treatments, maximum LWP was achieved in N1 (− 2.1 Mpa), which did differ with N0 (− 1.97 Mpa) treatment (Fig. 4).

**Figure 5:**
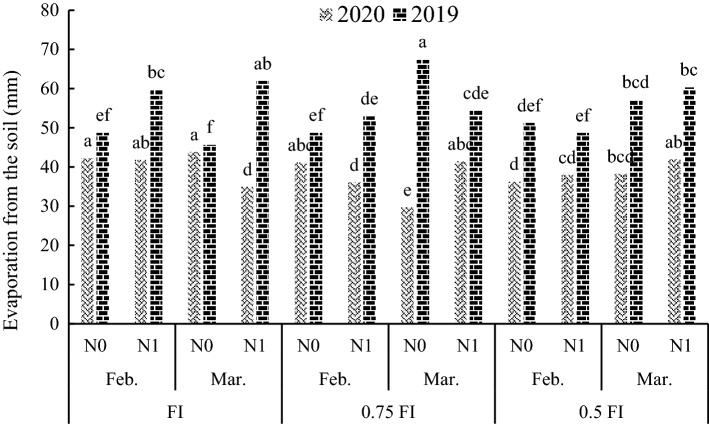
The effect of irrigation × sowing date × fertilizer on soil evaporation in two years. FI: full irrigation, N: nitrogen, Feb: February, Mar: March. Means with different letters did differ significantly.

Evaporation from soil affected by quadropule interaction of the treatmnants (Table 2). The results of the mean comparison showed that FI-late sowing-N0 treatment had the highest evaporation from the soil in 2020 (Fig. 5). In 2019, evaporation from soil was higher in the 0.75FI in late sowing and without nitrogen fertilizer treatment compared with other treatments.

**Figure 6:**
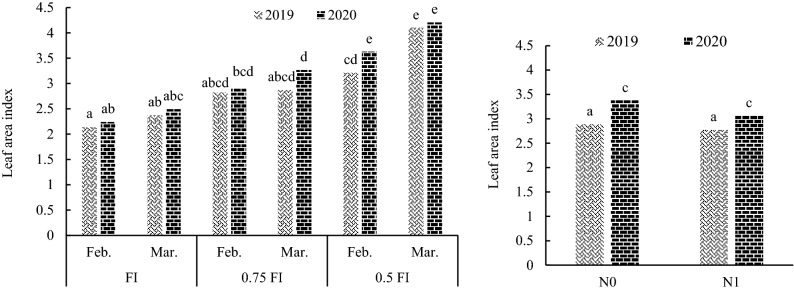
The interaction of irrigation × sowing date × year (**A**), and the nitrogen fertilizer main effect (averaged over irrigation and sowing treatments) on leaf area index (**B**) in 2019 and 2020 in pea. FI: full irrigation, N: nitrogen, Feb: February, Mar: March. Means with different letters did differ significantly.

Leaf area index (LAI) was affected by the effect of nitrogen fertilizer. Mean comparison for I × D effects for LAI indicated that no significant differences were identified between two sowing dates in irrigation regimes in both seasons (Fig. 6). The results showed that LAI increased significantly as the severity of water deficit conditions increased. Mean comparison for nitrogen main effect revealed that N0 presented higher LAI than N1 in both years (Fig. 6).

**Figure 7:**
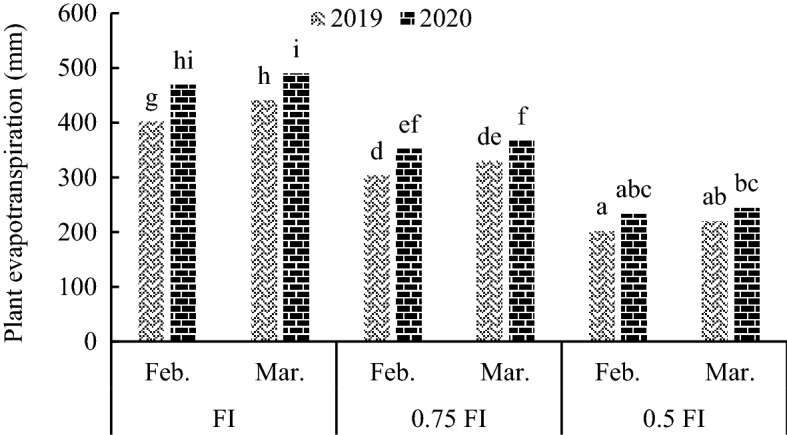
The effect of irrigation × sowing date × year on plant evapotranspiration. FI: full irrigation, Feb: February, Mar: March. The data averaged over nitrogen treatments. Means with different letters did differ significantly.

Plant evapotranspiration (ET) was affected by triple interaction of irrigation, sowing date and year. The results of the mean comparison for I × D × Y interaction indicated that the FI and late sowing treatment had higher ET in both years and that ET decreased as the severity of water defict irrigation increased (Fig. 7).

### Grain protein

**Figure 8:**
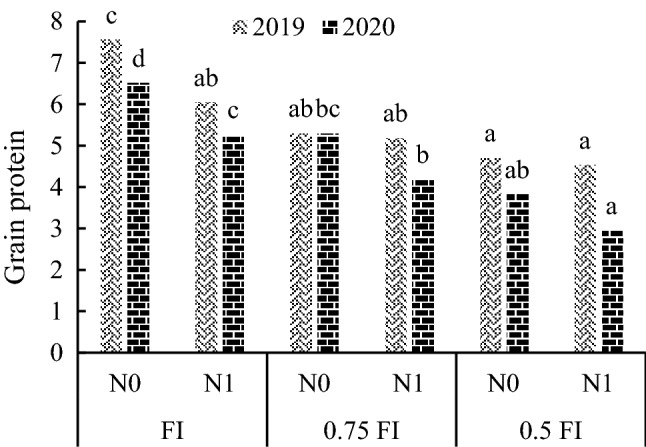
The effect of irrigation × nitrogen fertilizer interaction (the data averaged over sowing treatments) on grain protein (%) in pea. FI: full irrigation, Feb: February, Mar: March. Means with different letters did differ significantly.

The results of ANOVA showed that year, irrigation and nitrogen main effects and irrigation × nitrogen interaction were significant for grain protein. The highest grain protein that was observed in the FI-N0 treatment did differ with protein in FI-N1 treatment in two seasons (Fig. 8).

## Discussion

Ecological and nutritional managements including timely sowing, sufficient irrigation water along with application of a suitable amount of nitrogen help to improve growth and grain yield in crops^26^. Results of our study revealed that nitrogen and sowing date had significant effects of pea growth and that not all traits were affected by interactions of treatments suggesting heterogeneity in response of pea to the applied treatments. Heterogeneity in response of pea to the applied treatments demonstrated that management for sowing date, nitrogen fertilizer and irrigation regime could help for better growth and higher grain yield in regions with limited access to irrigation water at the reproductive stages of pea. Our results showed that peas had better growth in the the late sowing treatment. Vegetation cover, leaf water related chacarters, chlorophyll content, soil and plant ET and grain yield traits were higher in the late sown peas in at least one of irrigation treatments demosntrating an appropriate sowing time helps to mitigate the adverse effects of environmental conditions on crop productivity. Temperature, day length, percipitation and soil moisture differ in cultivations with different sowing times that affect plant growth and yield during the growing season^27^. Higher yield in pea in the March sowing date treatment compared to February sowing could be related to the lower risk of early frosting or cold weather damages in peas.

Results of the current study showed that irrigation water affects growth and grain yield in pea. Grain yield and yield related traits in the full irrigation (FI) and 0.75FI treatments of the late sown pea showed that 25% irrigation water can be saved without significant reduction in yield in regions where availability of water is limited. Evaluation of yield related traits such as the number of pods per plant showing simpler genetic control than grain yield per se might be helpful for the improvement of grain yield under water limited conditions^28–31^. Water use efficiency in 0.75FI and 0.5FI treatments did not differ with WUE in full irrigation in late sown peas which was in agreement with results of grain yield in deficit irrigation treatments. Optimal water use effieciency is one of the basic indicators of sustainable agriculture^32^. Under water deficient condition, the plant produces more yield compared to favorable moisture conditions^32^. Al-Barrak et al.^33^ reported that a decrease in water use efficiency will increase stress intensity that was inconsistent with the results of our study.

Although application of nirtogen is pivotal for the growth of crop plants, pea did not respond to nitrogen in our study. To maintain crop economical yield, it is necessary to optimize application of nitrogen for improvement of nitrogen use efficiency of plants reducing environmental risks such as impaired water quality, greenhouse effect and soil contamination^16^. Results of our study showed that nitrogen had inconsistent effects on pea traits. Previous studies have shown that the addition of nitrogen to pea has been disputed^34,35^. The application of nitrogen fertilizer did not show benefit to the yield traits in pea in our study. The N0 treatment of our study showed higher LAI, ET, grain weight and protein than application of nitrogen. In a study, nitrogen significantly affected the grain protein and yield in pea^34^. However, in the Achakzai et al.^35^ a significant negative correlation was found between the amount of nitrogen fertilizer and grain protein. It has been shown that a lower dose of nitrogen fertilizer effectively increases the rate of nitrogen remobilization compared to a high dose in pea (*P. sativum* L.)^16^.

Plant and soil evaporation are important parameters for evaluation of the adverse effects of drought stress. Results of our study showed that plant ET was lower in 0.5FI than in 0.75FI and full irrigation treatments whilst the highest soil evaporation achieved in FI, March sowing and without application of nitrogen treatment combination in 2020. In fact, in the first year more water molecules were exposed to evaporation from the soil surface, while in the second year high evaporation from the soil occurred in irrigation treatments with lower water supply. This could be due to the influence of other factors such as relative humidity, temperature and radiation which shows managing evaporation from the soil surface is not easy as it has been affected by various weather and soil factors^36^. In arid and semi-arid regions, temperature and solar radiation play a major role in evapotranspiration^37^.

## Conclusions

Results of this study showed that irrigation regimes, sowing date and application of nitrogen fertilizer significantly affected leaf area, water status and yield traits in pea. Adding nitrogen to the soil did not improve grain yield significantly and reduced protein content suggesting without application of nitrogen treatment could help sustainable agriculture. However, a short delay in sowing from February to March improved leaf water potential and increased yield related traits and water use efficiency in pea. Of the watering regimes tested, 0.75 and 0.5 field capacity (FC) treatemnts showed non-significant differences with full irrigation regimes (100% FC) for leaf water status and grain yield traits whilst resulted in lower plant and soil evaporations which shows at least 25% irrigation water can be saved without significant reduction in yield. Overall, results of our study suggested that without application of nitrogen and late sowing might improve grain yield and protein and water use efficiency of pea for cultivation under water limited conditions.

## Supplementary Information

Supplementary Information.

## Data Availability

The datasets generated during and/or analyzed during the current study are available from the corresponding author on reasonable request.

## References

[1] Ashraf MI, Pervez MA, Amjad M, Ahmad R, Ayub M (2011). Qualitative and quantitative response of pea (*Pisum*
*sativum* L.) cultivars to judicious applications of irrigation with phosphorus and potassium. Pak. J. Life Soc. Sci..

[2] Peksen E, Peksen A, Bozoglu H, Gülümser A (2004). Comparison of fresh pod yield and pod related characteristics in pea (*Pisum*
*sativum* L.) cultivars sown in autumn and spring under Samsun ecological conditions. Turk. J. Agric. For..

[3] Ayyub, C. M., Ziaf, K., Pervez, M. A., Rasheed, M. A. S., & Akhtar, N. Effect of seed maturity and storability on viability and vigor in pea (*Pisum sativum* L.) seeds, in *Proceedings of International symposium on prospects of Horticultural Industry in Pakistan hosted by Institute of Horticultural Sciences.* University of Agriculture, Faisalabad (28–30 March) (2007).

[4] Nonnecke IL (1989). Vegetable production.

[5] Urbano G, Aranda P, Gómez-Villalva E, Frejnagel S, Porres JM, Frías J, Vidal-Valverde D, López-Jurado M (2003). Nutritional evaluation of pea (*Pisum*
*sativum* L.) protein diets after mild hydrothermal treatment and with and without added phytase. J. Agric. Food Chem..

[6] Esho KB, Al-Kumar MK, Jubrael JM (2014). Diallel analysis using Hayman method to study genetic architecture of yield and it's component in peas (*Pisum*
*sativum* L.). Diyala J. Agric. Sci..

[7] Johnston AM, Clayton GW, Lafond GP, Harker KN, Hogg TJ, Johnson EN, May WE, McConnell JT (2002). Field pea seeding management. Can. J. Plant Sci..

[8] Voisin AS, Salon C, Munier-Jolain NG, Ney B (2002). Quantitative effects of soil nitrate, growth potential and phenology on symbiotic nitrogen fixation of pea (*Pisum*
*sativum* L.). Plant Soil.

[9] Duzdemir O, Kurunc AHMET, Unlukara A (2009). Response of pea (*Pisum*
*sativum*) to salinity and irrigation water regime. Bulg. J. Agric. Sci..

[10] Rasaei A, Ghobadi ME, Ghobadi M (2012). Effect of supplemental irrigation and plant density on yield and yield components of peas (*Pisum*
*sativum* L.) in Kermanshah region. Afr. J. Agric. Res..

[11] Tilman D, Cassman KG, Matson PA, Naylor R, Polasky S (2002). Agricultural sustainability and intensive production practices. Nature.

[12] Subhan F (2018). Effect of time of nitrogen application on growth and yield of peas. Bulletin Penelitian Hortikultura.

[13] Mehrabi F, Sepaskhah AR (2018). Interaction effects of planting method, irrigation regimes, and nitrogen application rates on yiehd, water and nitrogen use efficiencies of winter wheat (*Triticum aestivum*). Int. J. Plant Prod..

[14] McKenzie RH, Middleton AB, Flore N, Bremer E (2004). Evapotranspiration efficiency of pea in south and central Alberta. Can. J. Plant Sci..

[15] Faligowska A, Kalembasa S, Kalembasa D, Panasiewicz K, Szymanska G, Ratajczak K, Skrzypczak G (2022). The nitrogen fixation and yielding of pea in different soil tillage systems. Agronomy J..

[16] Hu F, Tan Y, Yu A, Zhao C, Coulter JA, Fan Z, Yin W, Fan H, Chai Q (2018). Low n fertilizer application and intercropping increases N concentration in pea (*Pisum*
*sativum* L.) grains. Front. Plant Sci..

[17] Soltani, A. *Crop Production.* Course notes (Gorgan University of Agricultural Science and Natural Resources, Iran, 2009).

[18] Rodriguez-Lizana A, Carbonell R, Gonzalez P, Ordonez R (2010). N, P and K released by the field decomposition of residues of a pea-wheat-sunflower rotation. Nutr. Cycl. Agroecosyst..

[19] Adamsen FJ, Coffelt TA (2005). Planting date effects on flowering, seed yield, and oil content of rape and crambe cultivars. Ind. Crops Prod..

[20] Singh N, Kaur N, Rana JC, Sharma SK (2010). Diversity in seed and flour properties in field pea (*Pisum sativum*) germplasm. Food Chem..

[21] Ahmed B, Hasan AK, Karmakar B, Hasan MS, Akter F, Saha PS, Haq ME (2020). Influence of date of sowing on growth and yield performance of field pea (*Pisum*
*sativum* L.) genotypes. Asian Res. J. Agric..

[22] Ishtiaq M, Ara N, Rashid A (2001). Response of different pea cultivars to various planting dates under the agro-climatic conditions of Swat [Pakistan]. Sarhad J. Agric..

[23] Kirk PL (1950). Kjeldahl method for total nitrogen. Anal. Chem..

[24] Ezeagu IE, Petzke JK, Metges CC, Akinsoyinu AO, Ologhobo AD (2002). Seed protein contents and nitrogen-to-protein conversion factors for some uncultivated tropical plant seeds. Food Chem..

[25] SAS Institute Inc. SAS/ACCESS® 9.4 (SAS Institute Inc, 2013).

[26] Cakmak O, Ozturk L, Karanlik S, Ozkan HAKAN, Kaya Z, Cakmak I (2001). Tolerance of 65 durum wheat genotypes to zinc deficiency in a calcareous soil. J. Plant Nutr..

[27] Alessi J, Power JF, Zimmerman DC (1981). Effects of seeding date and population on water-use efficiency and safflower yield. Agron. J..

[28] Sarawat P, Stoddard FL, Marshall DR, Ali SM (1994). Heterosis for yield and related characters in pea. Euphytica.

[29] Maleki A, Heidari A, Siadat A, Tahmasebi A, Fathi A (2011). Effect of supplementary irrigation on yield, yield components and protein percentages of chickpea cultivars in Ilam, Iran. J. Crop Ecophysiol..

[30] Hosseini NM, Palta JA, Berger JD, Siddique KHM (2009). Sowing soil water content effects on chickpea (*Cicer*
*arietinum* L.): seedling emergence and early growth interaction with genotype and seed size. Agric. Water Manag..

[31] Leng G, Zhang X, Huang M, Asrar GR, Leung LR (2016). The role of climate covariability on crop yields in the conterminous United States. Sci. Rep..

[32] Shabiri S, Ghasemi Golazani K, Golchin A, Saba J (2006). Effect of water deficit on phenology and yield of three chickpea cultivars (*Cicer*
*arietinum* L.). J. Agric. Knowl..

[33] Al-Barrak KM (2006). Irrigation interval and nitrogen level effects on growth and yield of canola (*Brassica*
*napus* L.). Sci. J. King Faisal Univ..

[34] Krga I, Simić A, Dželetović Ž, Babić S, Katanski S, Nikolić SR, Damnjanović J (2021). Biomass and protein yields of field peas and oats intercrop affected by sowing norms and nitrogen fertilizer at two different stages of growth. Agriculture.

[35] Achakzai AKK, Bangulzai MI (2006). Effect of various levels of nitrogen fertilizer on the yield and yield attributes of pea (*Pisum*
*sativum* L.) cultivars. Pak. J. Bot..

[36] Zhang Y, Chiew FH, Peña-Arancibia J, Sun F, Li H, Leuning R (2017). Global variation of transpiration and soil evaporation and the role of their major climate drivers. J. Geophys. Res. Atmos..

[37] Shih SF (1984). Data requirement for evapotranspiration estimation. J. Irrig. Drain. Eng..

