# Supplementary material for: The interacting effects of irrigation, sowing date and nitrogen on water status, protein and yield in pea (Pisum sativum L.)
Source: Sci Rep. 2022 Sep 25;12:15978. doi: 10.1038/s41598-022-20216-5 (PMC9510127; doi:10.1038/s41598-022-20216-5)
Supplement: Supplementary file 1 — Supplementary Information. [file 41598_2022_20216_MOESM1_ESM.docx]

SUPL. Fig. 1. The layout of the experimental design
